# Frequency of CFTR, SPINK1, and Cathepsin B Gene Mutation in North Indian Population: Connections between Genetics and Clinical Data

**DOI:** 10.1155/2014/763195

**Published:** 2014-01-27

**Authors:** Shweta Singh, Gourdas Choudhuri, Sarita Agarwal

**Affiliations:** ^1^Department of Genetics, Sanjay Gandhi Post Graduate Institute of Medical Sciences, Lucknow 226014, India; ^2^Gastroenterology and Hepatobiliary Sciences, Fortis Memorial Research Institute, Gurgaon 122002, India

## Abstract

*Objectives*. Genetic mutations and polymorphisms have been correlated with chronic pancreatitis (CP). This study aims to investigate the association of genetic variants of cystic fibrosis transmembrane conductance regulator (CFTR) and serine protease inhibitor Kazal type 1 (SPINK-1) genes and Cathepsin B gene polymorphisms with CP and to associate genetic backgrounds with clinical phenotypes. *Methods*. 150 CP patients and 150 normal controls were enrolled consecutively. We analyzed SPINK-1 N34S and IVS3+2T>C gene mutations by PCR-restriction-fragment length polymorphism (RFLP). The identification of DF508, G551D, G542X, R117H, and W1282X mutations was carried out by ARMS-PCR. S549N mutation, IVS8 polyTn polymorphism, and Cathepsin B Lec26Val were analysed by PCR-RFLP, nested PCR, and PCR-RFLP plus sequencing, respectively. *Results*. We found a significant association of SPINK1 (N34S) gene polymorphism. IVS1−37T>C polymorphism shows linkage with 101A>G. 300 chromosomes belonging to the CFTR subgroup exhibited minor allele frequency of 0.04, 0.03, 0.03, 0.013, 0.006, and 0.02 for DF508, G452X, G551D, S549N, R117H, and IVS8 T5, respectively. Except for R117H and IVS8 T5 polymorphisms, all other mutations showed significant variation. *Conclusion*. Analysis of potential susceptibility variants is needed to support nature of the genes and environment in pancreatitis. This data may help establish genetic screening and prenatal setup for Indian population.

## 1. Introduction

Pancreatitis is inflammatory diseases characterized by multifactorial pathogeneses. Chronic pancreatitis is characterized by parenchymal changes including inflammation, fibrosis, and loss of exocrine and endocrine tissue. Chronic Pancreatitis (CP) occurs in association with exogenous and endogenous factors including alcohol intake, smoking habits, pancreas divisum, sphincter of Oddi dysfunction, and/or a genetic predisposition [1–5]. The familial clustering of TCP suggests that genetic defect may predispose to the disease. The environmental factors may be operative on the background of a genetic predisposition to pancreatitis [6].

Mutation in the cystic fibrosis gene is associated with idiopathic chronic pancreatitis when present in heterozygous state in association with other CFTR polymorphism [7]. Combined data from other studies indicated that ~18% of subjects with ICP had common CF-causing mutations, where as ~2% were compound heterozygotes who had CF-causing mutation plus a milder CFTR allele [8]. Data on CFTR variants in TCP are very limited. All 27 CFTR coding exons, including the 5T allele, were analyzed, and the study population consisted of only 18 Indian TCP patients [9]. Pancreas divisum as a pancreatic ductal anomaly was able to induce chronic pancreatitis as a result of relative outflow obstruction; this explains why subsets of patients develop chronic pancreatitis [10, 11]. Serine protease inhibitor Kazal type 1 (SPINK-1) gene encodes for a pancreatic secretory trypsin inhibitor, a protein that has a primary role in counteracting activated trypsin effects [12]. SPINK1 N34S is mostly found in patients without a family history of CP; 15–40% of patients with so-called idiopathic CP carry N34S on one allele or on both alleles [13]. Patients with TCP display, in contrast to trypsinogen alterations, a very high incidence of SPINK1 variants, which is markedly more pronounced than that reported from Western countries. In 2002, four different research groups investigated Indian and Bangladeshi patients suffering from TCP, and all reported a strong association with the SPINK1 p.N34S alteration [14–17]. Furthermore, common and rare variants were found to be associated with idiopathic CP. Loss-of-function alterations in CTRC could predispose to pancreatitis by diminishing its protective trypsin-degrading activity [18, 19]. Cathepsin B is presumed to perform an essential role in the intrapancreatic activation of digestive enzymes by mediating premature trypsinogen activation [20]. Lysosomal cysteine protease is composed of a dimer of disulfide-linked heavy and light chains, both produced from a single protein precursor. It is a member of the peptidase C1 family [21]. Mutations in the CTSB gene have been linked to tropical pancreatitis [22]. For most multifactorial diseases as pancreatitis study of susceptible gene variants along with environmental factors may predispose the disease. Genetic mutation along with exogenous factors and clinical features may support the disease progression. The aim of this study was to investigate the association of cystic fibrosis transmembrane conductance regulator (CFTR) and serine protease inhibitor Kazal type 1 (SPINK-1) gene and Cathepsin B gene mutation with chronic pancreatitis (CP) and to associate genetic backgrounds with clinical phenotype in these three conditions. 

## 2. Material and Methods

### 2.1. Patients

The study was approved by the local ethics committee. Informed consent was obtained from each enrolled subject. One hundred fifty CP patients were enrolled consecutively from 2009 to 2013. CP diagnosis was based on typical clinical laboratory tests (fecal elastase and/or chymotrypsin, pancreolauryl test, and measurement of fecal fat excretion) and on diagnostic imaging (X-ray/ultrasonography/CT: pancreatic calcii cations; ultrasonography/CT: dilatation and irregularities of pancreatic duct; ERCP and/or MRCP: irregular dilatation of pancreatic duct branches; dilatation of the main pancreatic duct; pancreatic stones). Control had no history of pancreatitis and they were age and sex matched. Blood samples for genetic analysis were taken from 150 healthy volunteers; 85 were male (56.66%) and 65 female (43.33%), mean age 47.3 ± 15.6 years from the same geographical region.

### 2.2. Methods

Genomic DNA was extracted and purified from whole blood using phenol-chloroform method. All polymerase chain reaction (PCR) amplification experiments were carried out in an automated PCR thermal cycler. The mutation analysis for CFTR gene mutations (ΔF508, G542X, G551D, R117H, and W1282X) was carried out by ARMS-PCR method using primers as described by Ferrie et al. [23]. S549N, 5T allele of Intron 8 was done by PCR-RFLP and Nested PCR, respectively. SPINK1 gene N34S and IVS3+2T>C mutation was done by PCR-RFLP and Cathepsin B gene mutation leu26Val by PCR and sequencing.

### 2.3. Exclusion and Inclusion Criteria

The inclusion criteria for TCP include (1) radiological evidence of pancreatic intraductal calcification, (2) nonalcoholic, and (3) no family history of pancreatitis. The exclusion criteria for TCP include (1) absence of any other etiological factors like alcoholism, hypercalcaemia, gall stones, and (2) presence of pancreatic cancer. The inclusion criteria for control include (1) age and sex matched control, (2) no history of pancreatitis, and (3) absence of any other cause for abdominal pain. The exclusion criteria for control include (1) presence of pancreatic cancer and (2) history of alcohol ingestion.

### 2.4. Statistics

The significance of association between disease and marker alleles was calculated by means of Pearson's *χ* ^2^ test with s.d. for tables with more than four cells. The data was statistically analyzed by SPSS software (version 15.0). Chi Square was performed to obtain the *P* value. Genotype and allele frequency were analyzed using the EpiInfo (version 3.4; Centers for disease control and prevention) software package. The observed genotype frequencies were compared with those calculated from the Hardy-Weinberg disequilibrium theory (*p* ^2^ + 2*pq* + *q* ^2^ = 1), where *p* is the frequency of the variant allele and *q* = 1 − *p*.

## 3. Results

### 3.1. Association of SPINK1 Gene Mutation with Chronic Pancreatitis Compared with Controls

SPINK1 screening was done in all 150 cases and 150 controls. The proportion of SPINK1 gene mutation was found significantly higher in cases (57%) as compared to controls (3.3%). Of the three polymorphism screened, the most common polymorphism N34S was found in 41.3% cases and 2% in controls, OR: 36.60, 95% CI: 11.13–120.29, *P*-value: 0.001. IVS3+2T>C was found in 10% of cases and 0.67% in controls, OR: 10.6, 95% CI: 1.34–84.26, *P*-value: 0.014 and IVS1−37T>C was found in 7.3% in cases as compared to 0.67% in controls, OR: 11.38, 95% CI: 1.45–88.75, *P*-value: 0.008. The results were shown in Table 1. Carrier's frequency of SPINK1 mutations was 88 (58.6%) compared to noncarriers frequency 62 (41.3%). Prevalence of mutations was higher in more than 50% of patients and was found to be strongly associated with chronic pancreatitis but the variability was observed in mutation status; 44% of N34S mutation was observed in TCP patients as compared to other 14% of mutation of SPINK1 alone a contributing factor. Biochemical investigations like lipid profile (triglycerides, total cholesterol, HDL cholesterol, LDL cholesterol, and VLDL cholesterol). The value of serum high-density lipoprotein (HDL) cholesterol is significant (*P*-0.002) as it showed comparably same values in lower range with lower values of HDL in carriers groups as compared to noncarriers. However the levels of serum triglyceride, total cholesterol, low-density lipoprotein (LDL) cholesterol, and very low-density lipoprotein (VLDL) had no significant association between two groups. Significant association was observed in serum lipase (*P*-0.012) as values were almost same between carriers and noncarriers group presented in Table 2.

### 3.2. Association of CFTR Gene Mutation with Chronic Pancreatitis Compared with Controls

CFTR screening was done in all 150 cases and 150 controls. The proportion of CFTR gene mutation was found significantly higher in cases (32.60%) as compared to controls (10%) (OR = 5.6, 95% CI = 3.13–10.21, *P* = 0.001). Of the 6 mutation and 1 polymorphism screened, the most common mutation delta F508 was found in thirteen (8.7%) cases and three (1.9%) in controls (OR = 4.65, 95% CI = 1.29–16.674, *P* = 0.020), G542X in ten (6.7%) cases and three (1.99%) in controls (OR = 3.5, 95% CI = 0.94–12.986, *P* = 0.085), G551D in nine (6%) cases and four (2.65%) in controls (OR = 2.30, 95% CI = 0.70–7.74, *P* = 0.25), R117H in two (1.3%) and one (0.66%) in controls (OR = 1.3, 95% CI = 0.597–2.98, *P* = 1), S549N in three (2%) and one (0.66%) in controls (OR = 1.5, 95% CI = 0.847–2.690, *P* = 0.622), and 5T/7T in three (2%) in cases and one (0.66%) in controls (OR = 2.2, 95% CI = 0.66–7.4, *P* = 0.004) shown in Table 3. However, the W1282X mutation was not identified in any of the studied subjects. None of the subjects were found to be homozygous for any of the genotyped mutation of CFTR. Carriers (*n* = 37) of CFTR gene mutations among cases were compared with noncarriers (*n* = 113). Carriers were defined as those subjects who were found to be heterozygous for any of the six mutations studied; whereas noncarriers were those subjects in which none of the mutations were identified. A detailed demographic and clinical profile of carrier's versus noncarriers is given in Table 4.

### 3.3. Association of CTSB Gene Polymorphism with Chronic Pancreatitis Compared with Controls

Among 150 cases, 95.3% (143) were of wild type genotype, 4.6% (7) were of heterozygous, and no homozygous mutation was found. Similarly in 150 controls, 99.3% (149) were of wild type genotype, 0.66% (1) were of heterozygous, and no homozygous mutation was found. The odds ratio was (OR: 1.7, 95% CI: 1.341–2.381, *P*-value: 0.667). The wild type and mutant allele were found to be 97.6% (293) and 2.3% (7) in cases, respectively. In controls wild type and mutant allele were found to be 99.6% (299) and 0.33% (1), respectively. The results are shown in Table 5. No association of genotype and allele frequency was observed in TCP cases.

## 4. Discussion

N34S is a missense mutation in which an A → G transition at nucleotide 101 in exon 3 results in asparagines to serine substitution at amino acid 34. N34S is the most common mutation identified with respect to tropical chronic pancreatitis in various populations [24]. In our study, N34S (heterozygous) mutation in 62 (41.3%) cases and 3 (2%) in controls was recorded which shows significant association of N34S with TCP risk (OR: 19.4, 95% CI: 1.06–356.05, *P*-value: 0.006). N34S heterozygous mutation was more prevalent in comparison to homozygous mutation. There was no phenotypic correlation found in relation to homozygous or heterozygous mutation. Thus, it seems that N34S SPINK1 mutation is not disease inducer rather it is disease modifier mutation. These results were in agreement with those reported by Matsubayashi et al. [25]. SPINK1 provides the first line of defense against prematurely activated trypsinogen by physically blocking the active site of trypsin.

The IVS3+2T>C  (c.194+2T>C) mutation is a loss of functional splicing mutation; it affects the consensus splicing donor site in intron 3 and may cause skipping of the entire exon 3, where the trypsin-binding site is located [26]. The phenotypic variability of the homozygous IVS3+2T>C mutation in SPINK1 gene in CP patients has been established by this group. However, the results of the present study indicated IVS3+2T>C (heterozygous) mutation in 10 (6.7%) CP cases and 1 (0.67%) in controls which shows significant association of IVS3+2T>C mutation with TCP risk. A sole report of mutated SPINK1 in a CP patient outside the Indian subcontinent exists [27]. The results of the present study revealed the occurrence of IVS1−37T>C (heterozygous) mutation in 11 (7.3%) cases and 1 (0.67%) in controls which showed significant association with CP risk. Threadgold et al. [28] have shown that N34S; IVS1–37T 1 C mutation was associated with familial, idiopathic, and autoimmune CP. In the present study, higher frequency of delF508 (heterozygous) mutation among CP cases 13 (8.7%) was recorded as compared to controls 3 (1.9%). The analysis of allele frequency of CP cases and controls in present investigation indicated significant difference in mutant allele frequency of CP cases than the controls whereas wild allele frequency remains same in both these groups. Midha et al. [29] in a case-control study observed strong genetic susceptibility of ΔF508 mutation with TCP. In another such study, a significant association of ΔF508 mutation was observed with TCP, the value of frequency being 11.8%. Cathepsin B is an enzymatic protein belonging to the peptidase (or protease) families. In humans, it is encoded by the CTSB gene. The protein encoded by this gene is a lysosomal cysteine protease composed of a dimer of disulfide-linked heavy and light chains, both produced from a single protein precursor [30]. The results of the present study indicated Leu26Val (heterozygous) mutation in 4.6% (7) TCP cases and 0.66% (1) in controls. These results reflected no association of Leu26Val with CP risk.

## 5. Conclusion

In pancreatitis the context includes the inflammatory response, clinical features, and exogenous factors. Molecular genetics variants modify the diseases status and have corelation with clinical background. Therefore, further studies among different populations with pancreatitis and well-defined clinical data will help to verify these preliminary results and attempt to characterize a possible influence of gene mutation in etiology of a chronic pancreatitis.

## Figures and Tables

**Table 1:** Genotype and allele frequency of SPINK1 gene polymorphisms.

Polymorphisms	Type	CP (*n* = 150) (%)	Cont (*n* = 150) (%)	OR (95% CI) *P* value
101A>G genotypes	Wild (AA)	83 (55.3)	147 (98)	Reference
	Heterozygous (AG)	62 (41.3)	03 (2)	36.60 (11.13–120.29) 0.001*
	Mutant (GG)	05 (3.3)	0	19.43 (1.06–356.05) 0.006*

101A>G alleles	Wild allele (A)	228 (76)	297 (99)	Reference
	Mutant allele (G)	72 (24)	3 (1)	31.26 (9.72–100.53) 0.001*

IVS3+2T>C genotypes	Wild (TT)	140 (93.3)	149 (99.3)	Reference
	Heterozygous (TC)	10 (6.7)	1 (0.67)	10.6 (1.34–84.26) 0.014*

IVS3+2T>C alleles	Wild allele (T)	290 (96.7)	299 (99.7)	Reference
	Mutant allele (C)	10 (3.3)	1 (0.3)	10.3 (1.31–81.0) 0.014*

IVS1−37T>C genotypes	Wild (TT)	139 (92.7)	149 (99.3)	Reference
	Heterozygous (TC)	11 (7.3)	1 (0.67)	11.38 (1.45–88.75) 0.008*

IVS1−37T>C alleles	Wild allele (T)	289 (96.3)	299 (99.7)	Reference
	Mutant allele (C)	11 (3.7)	1 (0.3)	11.38 (1.45–88.7) 0.008*

CP: chronic pancreatitis, Cont: control, *significant association (*P* < 0.05).

**Table 2:** Demographic and clinical profiles of 150 chronic pancreatitis cases (carriers versus noncarriers) screened for SPINK1 gene.

Characteristics	Carriers (88)	Noncarriers (62)	*P* value
Age in months (M ± SD)	33.9 ± 11.7	32.3 ± 11.6	0.395
Weight in kg (M ± SD)	56.0 ± 13.3	52.2 ± 12.8	0.082
BMI (M ± SD)	21.2 ± 4.6	20.1 ± 4.5	0.132
FBS (M ± SD)	56.2 ± 102.6	48.2 ± 103.8	0.567
PPBS (M ± SD)	153.6 ± 79.3	168.9 ± 68.5	0.210
HbA1C (M ± SD)	5.93 ± 1.7	5.23 ± 1.5	0.225
Triglyceride (M ± SD)	118.2 ± 92.0	116.3 ± 59.56	0.883
Total cholesterol	156.1 ± 51.29	162 ± 49.3	0.381
HDL-cholesterol	35.8 ± 8.82	76.3 ± 9.86	0.002*
LDL-cholesterol	86.2 ± 32.2	88.8 ± 28.7	0.432
VLDL-cholesterol	26.9 ± 12.41	17.5 ± 7.77	0.012*
Serum amylase	1023 ± 488.8	727.7 ± 480	0.013*
Serum lipase	732.40 ± 452.2	888.9 ± 420	0.112
Serum calcium	8.9 ± 1.2	9.11 ± 1.09	0.226

*Mean ± SD (*significant association found).

**Table 3:** Genotype and allele frequency of CFTR gene.

Polymorphism	Type	CP (*n* = 150) (%)	Cont (*n* = 150) (%)	OR (95% CI) *P* value
ΔF508 genotypes	Wild (FF)	137 (91.3)	147 (98)	Reference
	Heterozygous (FT)	13 (8.7)	03 (1.9)	4.65 (1.29–16.67) 0.020*

ΔF508 alleles	Wild allele (F)	287 (95.7)	297 (99)	Reference
	Mutant allele (T)	13 (4.3)	3 (1)	4.4 (1.26–15.906) 0.022*

G542X genotypes	Wild (GG)	140 (93.3)	147 (98)	Reference
	Heterozygous (GT)	10 (6.7)	3 (2)	3.5 (0.94–12.98) 0.085

G542X alleles	Wild allele (G)	290 (97)	297 (99)	Reference
	Mutant allele (T)	10 (3.3)	3 (1)	3.4 (0.929–12.53) 0.0925

G551D genotypes	Wild (GG)	141 (94)	146 (97.3)	Reference
	Heterozygous (GA)	9 (6)	4 (2.7)	2.3 (0.70–7.74) 0.25

G551D alleles	Wild allele (G)	291 (97)	296 (98.6)	Reference
	Mutant allele (A)	9 (3)	4 (1.3)	2.28 (0.69–7.51) 0.26

R117H genotypes	Wild (RR)	148 (98.6)	149 (99.3)	Reference
	Heterozygous (RH)	2 (1.33)	1 (0.66)	1.3 (0.597–2.98) 1.0

R117H alleles	Wild allele (R)	298 (99.3)	299 (99.6)	Reference
	Mutant allele (H)	2 (0.66)	1 (0.33)	1.3 (0.597–2.98) 1.0

S549N genotypes	Wild (GG)	147 (98)	149 (99.3)	Reference
	Heterozygous (GA)	03 (2)	01 (0.66)	1.5 (0.847–2.690) 0.6225

S549N alleles	Wild allele (G)	297 (99)	299 (99.6)	Reference
	Heterozygous (A)	3 (1)	1 (0.3)	1.5 (0.849–2.666) 0.623

IVS8−T_n_ genotypes	Wild Type (7T/7T, 9T/7T, 9T/9T)	138 (92)	147 (98)	Reference
	Heterozygous (7T/5T, 9T/5T)	11 (7.3)	03 (2)	3.90 (1.067–14.301) 0.03*
	Mutant (5T/5T)	01 (0.66)	0	

IVS8−T_n_ alleles	Wild allele (7T, 9T)	284 (94.6)	297 (99)	Reference
	Mutant allele (5T)	13 (8.6)	03 (1)	1.9 (1.60–2.246) 0.003*

CP: chronic pancreatitis, Cont: control, *significant association (*P* < 0.05).

**Table 4:** Demographic and clinical profile of 150 chronic pancreatitis cases (carriers versus noncarriers) screened for CFTR gene.

Characteristics	Carriers (49)	Noncarriers (101)	*P* value
Age in months (M ± SD)	36.1 ± 11.63	31.8 ± 11.5	0.036
Weight in kg (M ± SD)	57 ± 12.7	53.2 ± 13.3	0.126
BMI (M ± SD)	21.2 ± 4.21	20.5 ± 4.77	0.309
FBS (M ± SD)	117 ± 58.5	96.3 ± 48.6	0.022*
PPBS (M ± SD)	174.3 ± 86.16	153.3 ± 68.4	0.109
HbA1C (M ± SD)	5.29 ± 1.7	5.65 ± 1.65	0.223
Triglyceride (M ± SD)	134.8 ± 115.0	108.9 ± 53.4	0.032*
Total cholesterol (M ± SD)	165.5 ± 46.3	155.3 ± 52.1	0.228
HDL-cholesterol (M ± SD)	36.1 ± 9.75	60.7 ± 13.5	0.032
LDL-cholesterol	87.2 ± 25.9	84.8 ± 40.3	0.852
VLDL-cholesterol	28.3 ± 11.2	22.3 ± 13.4	0.296
Serum amylase	607.2 ± 540.0	722.7 ± 465.7	0.179
Serum lipase	825.4 ± 639.1	569.5 ± 443.1	0.035*
Serum calcium	9.06 ± 0.944	8.9 ± 1.3	0.631

*Mean ± SD (*significant association found).

**Table 5:** Genotype and allele frequency of C76G polymorphism.

Polymorphisms	Type	CP (*n* = 150) (%)	Cont (*n* = 150) (%)	OR (95% CI) *P* value
C76G genotypes	Wild (CC)	143 (95.3)	149 (99.3)	Reference
	Heterozygous (CG)	7 (4.6)	1 (0.66)	1.7 (1.341–2.381) 0.667*

C76G alleles	Wild allele (C)	293 (97.6)	299 (99.6)	Reference
	Mutant allele (G)	7 (2.3)	1 (0.33)	1.7 (1.344–2.326) 0.0685*

CP: chronic pancreatitis, Cont: control, *no association (*P* < 0.05).
